# Graph theoretical analysis of resting magnetoencephalographic functional connectivity networks

**DOI:** 10.3389/fncom.2013.00093

**Published:** 2013-07-12

**Authors:** Lindsay Rutter, Sreenivasan R. Nadar, Tom Holroyd, Frederick W. Carver, Jose Apud, Daniel R. Weinberger, Richard Coppola

**Affiliations:** ^1^MEG Core Facility, National Institute of Mental HealthBethesda, MD, USA; ^2^Clinical Brain Disorders Branch, National Institute of Mental HealthBethesda, MD, USA; ^3^Lieber Institute for Brain DevelopmentBaltimore, MD, USA

**Keywords:** schizophrenia, small world, magnitude squared coherence, clustering coefficient, path length, exponentially truncated power-law, synthetic aperture magnetometry, default network

## Abstract

Complex networks have been observed to comprise small-world properties, believed to represent an optimal organization of local specialization and global integration of information processing at reduced wiring cost. Here, we applied magnitude squared coherence to resting magnetoencephalographic time series in reconstructed source space, acquired from controls and patients with schizophrenia, and generated frequency-dependent adjacency matrices modeling functional connectivity between virtual channels. After configuring undirected binary and weighted graphs, we found that all human networks demonstrated highly localized clustering and short characteristic path lengths. The most conservatively thresholded networks showed efficient wiring, with topographical distance between connected vertices amounting to one-third as observed in surrogate randomized topologies. Nodal degrees of the human networks conformed to a heavy-tailed exponentially truncated power-law, compatible with the existence of hubs, which included theta and alpha bilateral cerebellar tonsil, beta and gamma bilateral posterior cingulate, and bilateral thalamus across all frequencies. We conclude that all networks showed small-worldness, minimal physical connection distance, and skewed degree distributions characteristic of physically-embedded networks, and that these calculations derived from graph theoretical mathematics did not quantifiably distinguish between subject populations, independent of bandwidth. However, *post-hoc* measurements of edge computations at the scale of the individual vertex revealed trends of reduced gamma connectivity across the posterior medial parietal cortex in patients, an observation consistent with our prior resting activation study that found significant reduction of synthetic aperture magnetometry gamma power across similar regions. The basis of these small differences remains unclear.

## Introduction

The human brain is a complex biological system composed of many interacting subsystems, and its collective behavior cannot simply be understood in terms of its isolated components (Varela et al., 2001). Viewing the brain as a complex network has motivated the recent shift from pinpointing local activations of cortex to identifying widespread functional networks. One common measurement used in the latter approach is functional connectivity, or the statistical interrelations between physiological time series recorded from different brain areas, which is assumed to reflect functional interactions.

Graph theoretical analysis can be used to characterize complex patterns of functional connectivity from a network perspective. A graph *G* = (*V*, *E*, *W*) is a mathematical description of a network, which is essentially reduced to a collection of nodes (vertices, *V*) connected by lines (edges, *E*) that hold values (weights, *W*). The presence of an edge in a graph that represents functional brain networks indicates functional connectivity between the brain sources (vertices) it links.

Modern magnetoencephalography (MEG) is an ideal method to study complex brain systems because it covers the whole head with a large number of sensors that provide measurements on the time scale of cognitive processes. In this study, we explored MEG functional networks of the so-called resting human brain in health and schizophrenia by means of graph theory. Two graphical tools were of particular interest to us, *small worldness* and *degree distribution*.

The small world phenomenon, also commonly called six degrees of separation, was first observed in social networks in which the number of intermediate acquaintances between any two people is surprisingly small (Milgram, 1967). Although small worldness and its theories were introduced more than four decades ago, the small world phenomenon was only recently translated to a more quantifiable physical basis in an algorithm proposed by Watts and Strogatz (Watts and Strogatz, 1998; Watts, 1999).

They constructed a computational model of a perfectly ordered graph, in which each node was directly connected to its four nearest neighbors. This lattice topology demonstrated high clustering between nearby nodes because the path length (number of intermediary edges) between them was small by design. In contrast, the path length between distant nodes (on opposite ends of the lattice) was large, rendering the minimum path length averaged across all possible pairs of nodes as also large. They then randomly rewired all edges of the lattice until it had been transformed into a perfectly random network which had theoretical values of low clustering and low average minimum path length.

Importantly, however, they found that if they only introduced relatively few random rewirings into the lattice, it would mediate a short average minimum path length that was, for the most part, undetected at the local level. Hence, they found a class of graphs that were topologically intermediate between ordered and random graphs, exhibiting both the dense local interconnectedness observed in lattices and the high global integration (low average minimum path length) observed in random networks, which they called small world networks in allusion to the small world phenomenon.

Following this quantitative demonstration that a few short-cuts can have significant impact on network topology, small worldness has been reported in a range of complex networks ranging from metabolic systems and food webs to transportation systems and electrical power grids (Strogatz, 2001; Latora and Marchiori, 2003; Grigorov, 2005).

There are theoretical and empirical motives behind pursuing a small world analysis of human brain networks. To start with, several groups have argued that optimal brain functioning requires an appropriate balance between local specialization and global integration of brain activity (Tononi et al., 1998; Sporns et al., 2000; Latora and Marchiori, 2001; Le van Quyen, 2003), which suggests that brain networks might exhibit small world properties of high clustering (consistent with modular/segregated processing) and low average minimum path length (compatible with distributed/integrated processing). Moreover, the brain is a complex network since it must balance these two opposing forces (Tononi et al., 1998; Sporns et al., 2000), and therefore might show small world characteristics given the widespread observation of small worldness in so many other complex systems.

Small world topology might also represent an optimal brain organization for synchronization robustness between different brain regions (Watts and Strogatz, 1998; Lago-Fernandez et al., 2000; Latora and Marchiori, 2001; Barahona and Pecora, 2002; Masuda and Aihara, 2004). For instance, non-identical Hodgkin-Huxley neurons coupled with excitatory synapses show coherent oscillations in regular graphs, fast response in random graphs, and both coherent and fast responses in small world graphs (Lago-Fernandez et al., 2000). Importantly, synchronization of neural activity denotes physiological mechanisms of functional integration (Singer, 1999; Varela et al., 2001; Fries, 2005; Yu et al., 2008), and in this manner, small world networks of the brain allow for efficient information processing (Latora and Marchiori, 2001; Mathias and Gopal, 2001; Sporns and Zwi, 2004) and learning (Simard et al., 2005), as well as conditional robustness against malfunctioning brain regions (Albert et al., 2000; Achard et al., 2006).

Along these lines, it has been proposed that conservation of wiring cost has been an important selection pressure on the evolution of human brain components (Durbin and Mitchison, 1990; Chklovskii et al., 2002) since longer axonal projections are metabolically and materially expensive (Cherniak, 1994). With that said, small world architecture derived from anatomical and functional human brain connectivity models has been thought to deliver an economic strategy of maximizing global and local efficiency while minimizing axonal wiring connections (Latora and Marchiori, 2003; Achard and Bullmore, 2007; Humphries et al., 2007; Bassett et al., 2008).

A small, but growing, number of studies have confirmed small world features in healthy functional human brains when engaged in no proscribed activity (i.e., “rest”), and the evidence has been consistent (Stam, 2004; Eguiluz et al., 2005; Salvador et al., 2005; Achard et al., 2006; Bassett et al., 2006; Micheloyannis et al., 2006; Achard and Bullmore, 2007; Liu et al., 2008; Van den Heuvel et al., 2008). Remarkably, these studies reached the same conclusion albeit their diverse range of functional connectivity estimations (synchronization likelihood, wavelet decomposition, and partial correlation), nodal dimensions (macroscopic/regional and mesoscopic/voxel), and neuroimaging modalities [functional magnetic resonance imaging (fMRI), electroencephalography (EEG), and MEG]. (For more generalized reviews of healthy brain networks organized as small worlds, both functionally and structurally, in various modalities and testing conditions, see He and Evans, 2010; Sporns, 2011).

The optimal small world patterns seen in healthy functional brain networks may be disrupted in brain diseases: Brain tumors (Bartolomei et al., 2006), Alzheimer's disease (Stam et al., 2007a, 2009; Xie and He, 2012), epilepsy (Ponten et al., 2007, 2009), multiple sclerosis (Schoonheim et al., 2011) and schizophrenia (Micheloyannis et al., 2006; Liu et al., 2008; Rubinov et al., 2009) have all exhibited atypical small world functional brain topologies. To date, however, reports of how or even whether small world properties are disturbed in some of these clinical conditions have remained largely inconsistent (For recent reviews, see Reijneveld et al., 2007; Stam and Reijneveld, 2007; Van den Heuvel and Hulshoff Pol, 2010).

The second graphical approach often used to probe the functional organization of brain networks is degree distribution *P*(*k*). One of the most basic descriptions of a vertex is its degree *k*, or the number of edges that connect it to the rest of the graph. The average degree of the network is then *k*_net_. Highly connected vertices have large degrees and are often interpreted to function as network hubs. The degree distribution of a graph is the fraction of nodes with degree *k*, and can be used to assay the hierarchy of potential hubs in the network.

Efforts to classify the degree distribution of healthy functional brain networks have been less conclusive than those of small worldness, with some studies reporting scale-free organizations (Eguiluz et al., 2005; Van den Heuvel et al., 2008) and others reporting exponentially truncated power-law distributions (Achard et al., 2006; Bassett et al., 2006). In any case, these studies all suggest non-random degree distributions of resting healthy functional brain networks that are, at least in part, compatible with the occurrence of hubs.

One goal of this study was to determine whether source-localized magnetoencephalographic functional connectivity brain networks in resting health would show, across one or more bandwidths, non-random degree distributions and small world properties. We could not hypothesize whether graph theoretical measurements in this study would distinguish between healthy controls and schizophrenic patients because the topic remains elusive in the literature (Reijneveld et al., 2007; Stam and Reijneveld, 2007; Bassett et al., 2008; Bullmore and Sporns, 2009; Van den Heuvel and Hulshoff Pol, 2010). However, we recently found that resting patients showed significant reduction of source-localized MEG gamma power in the posterior medial parietal cortex, and wanted to determine whether similar differences are also present from functional connectivity perspectives, using datasets and preprocessing techniques as consistent as possible to our original study (Rutter et al., 2009).

## Materials and methods

### Subjects

We initially wanted to use the same datasets (38 healthy controls and 38 patients) we applied in our previous resting activation study (Rutter et al., 2009). However, functional connectivity measurements presented technical limitations that led us to choose a subset of 40 datasets from the original 76; further explanation of this selection process is provided later. The age-gender matched sample used for this study included 20 patients (6 females, 14 males; mean age: 31.2 ± 10.9, age range: 20.7–48.6) and 20 healthy controls (6 females, 14 males; mean age: 31.3 ± 10.8, age range: 21.3–54.2), all right handed (Oldfield, 1971).

Data was collected as part of the Clinical Brain Disorders Branch Genetic Study of Schizophrenia (National Institutes of Health Study ID NCT00001486, DR Weinberger, PI) and subject screening procedures were approved by the National Institute of Mental Health Institutional Review Board. Details of the criteria used to screen our subjects can be found elsewhere (Egan et al., 2000). All subjects with the diagnosis of schizophrenia were receiving antipsychotic drugs.

### Data acquisition

Participants were instructed to rest with eyes closed in a lit, magnetically shielded room for a 4 min recording. MEG signals were continuously recorded with a 275-gradiometer SQUID sensor array over the inner surface of a whole-head helmet (the former CTF Systems, Coquitlam BC, Canada). Anatomical MRI (3T General Electric MRI scanner) and MEG data were registered onto a common coordinate system for each subject using three fiducial references. All datasets in this study were also used in our previous study; see (Rutter et al., 2009) for further description of acquisitional logistics.

### Preprocessing

An identical preprocessing pipeline was applied to each of the potential 76 datasets: First, raw neuromagnetic data was digitized at a sampling rate of 600 Hz (bandwidth of 0–150 Hz) and filtered online in synthetic third gradient mode for background noise reduction. A 42,000 sampled (70 s) epoch, with minimal eye artifacts and head movement less than 0.5 cm, was selected off-line from each dataset. The data were then broadband filtered (1–80 Hz), and a minimal high-pass filter (0.61 Hz) and powerline filter (60 Hz) were used in addition to direct current offset.

Three-dimensional source projection of a given dataset onto a standardized brain template resulted in a whole-brain grid that contained 3291 cubic voxels with 7.5 mm width dimensions. For each dataset, MEG signals were translated to source weights for the 3291 voxels using single-state pseudo Z-deviate synthetic aperture magnetometry (SAM) (Vrba and Robinson, 2001). From the magnetic fields recorded by the 275 sensors, SAM generates a unique beamformer (275×1 vector of weighting factors) for each voxel in the cortex. Volumetric representation of brain activity was hence given in the form of 3291 virtual channels (linear combinations of measurements over time).

Minimum-variance beamforming estimates current dipole power changes in voxels across particular time windows and frequency bands. Optimal orientation of dipoles was estimated using the vector based method of Sekihara et al. Sekihara et al. (2001). The power source distribution of our SAM imaging was normalized with a constant noise estimate. An array ∑ of the *N* = 275 sensors was constructed with a constant noise variance *v*_θ_, defined as the smallest eigenvalue of the covariance matrix:

(1) $$\sum =\left(\begin{array}{cccc} v_{1}^{2} & \cdots & \cdots & 0\\ \vdots & v_{2}^{2} & \cdots & \vdots \\ \vdots & \vdots & \ddots & \vdots \\ 0 & \cdots & \cdots & v_{N}^{2} \end{array}\right)$$

The estimated sensor noise ${\hat{v}}_{\theta }^{2}$ was calculated as:

(2) $${\hat{v}}_{\theta }^{2}=H_{\theta }^{T}\sum H_{\theta },$$

where *H*_θ_ represents the *N* × 1 unique beamformer generated for each voxel in the cortex. The estimated source power ${\hat{S}}_{\theta }^{2}$ can then be calculated as:

(3) $$\begin{array}{l} {\hat{S}}_{\theta }^{2}={\left(H_{\theta }^{T}X\right)}^{2}\\ \;=\left(H_{\theta }^{T}X\right){\left(H_{\theta }^{T}X\right)}^{T}\\ \;=\left(H_{\theta }^{T}X\right)\left(H_{\theta }^{T}X^{T}\right),\\ \;=H_{\theta }^{T}\left(XX^{T}\right)H_{\theta }\\ \;=H_{\theta }^{T}CH_{\theta } \end{array}$$

where matrix *X* consists of rows containing data points for the *N* = 275 sensor channels and columns containing the sensor values, and covariance matrix *C* represents the covariance between sensor channels in *X* after the removal of the mean from each channel. The normalized estimated power in the voxel is then a ratio of the estimated source power and estimated noise variance of the voxel:

(4) $$Z_{\theta }^{2}=\frac{{\hat{S}}_{\theta }^{2}}{{\hat{v}}_{\theta }^{2}}=\frac{H_{\theta }^{T}CH_{\theta }}{H_{\theta }^{T}\sum H_{\theta }}$$

### Defining network nodes

The 3291 source-localized virtual channels for each subject were converted to ASCII format, down-sampled from 600 to 200 Hz, and filtered into four narrower bandwidths as per classic electrophysiology (Θ: 4–8 Hz, α: 8–14 Hz, β: 14–30 Hz, γ: 30–80 Hz).

We initially generated source weights for all 3291 voxels within each of the 76 potential datasets. However, when estimating source activity across such a large set of voxels, we inevitably found voxels with low signal-to-noise ratios (S:N ≤ 1), where the quantity and location of such voxels varied across individual datasets. For accuracy, we only rendered a voxel usable if S:N > 1 for all datasets.

Because of this constraint, and the fact that graph analyses performed at the individual level are computationally expensive, we chose 40 datasets that maintained the highest number of usable voxels (2872) as well as the age-gender match between patients and controls. Discarded voxels were dispersed throughout the brain and were not confined to any particular gyrus or hemisphere. This routine concluded with 40 datasets that each contained 2872 voxel (node) time series, each with 14,000 samples, across four distinct bandwidths (Θ, α, β, γ).

### Estimating association between nodes

Magnitude squared coherence, a generalization of correlation to the frequency domain, was computed between each pair of voxels in each dataset as a measure of linear relationship. From the time series of voxels *i* and *j*, ${\hat{x}}_{i}^{\left(t\right)}$ and, ${\hat{x}}_{j}^{\left(t\right)}$ we used Fourier transformation to obtain complex frequency-domain representations, *x*^(*f*)^_*i*_ and *x*^(*f*)^_*j*_, and calculated the cross power spectral density defined as:

(5) $$P_{ij}(f)\equiv x_{i}(f){\bar{x}}_{j}(f)$$

in which the overbar symbolizes complex conjugation. Complex-valued coherence is a function of the cross power spectral density and power spectral densities of *i* and *j*:

(6) $$C_{ij}(f)\equiv \frac{P_{ij}(f)}{\sqrt{P_{ii}(f)P_{jj}(f)}}$$

and magnitude squared coherence is the absolute value of complex-valued coherence squared:

(7) $$Coh_{ij}(f)\equiv {\left|C_{ij}(f)\right|}^{2}$$

For clarification, what we hereafter refer to as coherence is simply magnitude squared coherence. Coherence estimates how well *i* and *j* correspond at a specific frequency without a component of directionality; it ranges between 0 and 1. We obtained a single value of coherence for each voxel pair in each previously-filtered bandwidth (Θ, α, β, γ) by averaging the set of coherence values associated with the frequencies that constituted that bandwidth. For all calculations, we used the function *mscohere* in the Signal Processing Toolbox of MATLAB Software, using a Periodic Hamming window, sample overlap of 50%, and default FFT length.

Coherence remains one of the most studied tools for investigating interactions among neuron signals. It also forms some of the current mechanisms proposed for communication between brain regions (Fries, 2005). Recent work has also implicated that various phase value measures “provides equivalent information to the cross-correlation of the two complex time series” (Aydore et al., 2013). For these reasons, we believe coherence may be an appropriate tool in the time series we consider in the current work.

### Generating association matrices

These coherence values, believed to reflect inter-voxel functional connectivity, were represented as an association matrix *M*, where element *M*_*ij*_ contained the coherence value between voxels *i* and *j*. We repeated this process for each bandwidth, which resulted in four {2872 × 2872} matrices *M* for each subject.

From each association matrix *M*, we derived two thresholded matrices using an arbitrary threshold 0 ≥ τ ≥ 1: We produced a binary-valued adjacency matrix *A*, where *A*_*ij*_ = 1 if *M*_*ij*_ ≥ τ and *A*_*ij*_ = 0 if *M*_*ij*_ < τ, and a weighted matrix *W*, where *W*_*ij*_ = *M*_*ij*_ if *M*_*ij*_ ≥ τ and *W*_*ij*_ = 0 if *M*_*ij*_ < τ. For all indices *i* and *j*: *A*_*ij*_ = *A*_*ji*_, *W*_*ij*_ = *W*_*ji*_, *A*_*ii*_ = 0, and *W*_*ii*_ = 0.

### Constructing graphs

There are several variations of graphs: For example, graphs can be unweighted or weighted. An unweighted (binary) graph contains edge weights of either zero or unity. In contrast, when graded values are associated to edges, the corresponding graph is called a weighted graph, and its edge values can be used to indicate the strength of their relationships. A graph can also be undirected or directed: An undirected graph indicates symmetric edge relationships between its vertices (*E*_*ij*_ = *E*_*ji*_), whereas a directed graph signifies that its edges have causality (*E*_*ij*_ ≠ *E*_*ji*_).

We were able to abstract graphs *G*_*A*_ and *G*_*W*_, from matrices *A* and *W* respectively, by inputing lines between voxel pairs that held coherence values exceeding the threshold. Because we used coherence, a symmetric measurement, graph *G*_*A*_ was undirected and unweighted, and graph *G*_*W*_ was undirected and weighted. Repeating this process for each subject (40) and bandwidth (4) led to the formation of 160 sets of graphs *G*_*A*_ and *G*_*W*_ at the specified threshold. The dimensions of these 320 graphs were identical since we used the same number of nodes (*N* = 2872) for each subject.

### Computing small world metrics

We could next characterize possible small world properties in the undirected graphs that represented functional brain networks by calculating two key metrics of small worldness, the clustering coefficient *C* and the shortest path length *L*.

In some networks, if vertex *i* has edge connections to vertices *j* and *h* (*E*_*ij*_ = *E*_*ih*_ = 1), then it is also probable that *j* is adjacent to *h* (*E*_*jh*_ = 1); this phenomenon can be quantified with the clustering coefficient. The clustering coefficient of vertex *i*, 0 = *C*_*i*_ = 1, is simply a ratio of the number of existing links between its neighbors to the number of maximum possible links between its neighbors, with its neighbors being defined as nodes directly connected to i with one edge. Thus, for graph *G*_*A*_:

(8) $$C_{i}=\frac{2}{k_{i}(k_{i}-1)}\sum_{j\;\neq \;h\;\in \;V}E_{ij}E_{ih}E_{jh}$$

In the equation above, we divided the maximum number of possible neighborhood connections *k*_*i*_(*k*_*i*_ − 1) by two as we are observing undirected graphs. In an undirected but weighted graph, in our case *G*_*W*_, the clustering coefficient incorporates node-neighbor edge weights into the calculations:

(9) $$C_{i}=\frac{2}{W_{ki}(k_{i}-1)}\sum_{j\;\neq \;h\;\in \;V}\left(\frac{W_{Eij}+W_{Eih}}{2}\right)E_{ij}E_{ih}E_{jh}$$

where *W*_*ki*_ is the weighted degree of *i* and *W*_*Eij*_ and *W*_*Eih*_ are the weighted edges of *i* with *j* and *h*. Both binary and weighted clustering coefficients range between 0 and 1. The mean clustering coefficient of the graph, *C*_net_, is then determined by averaging nodal clustering coefficients across the entire network.

(10) $$C_{\text{net}}=\frac{1}{N}\sum_{i\;\in \;V}C_{i}$$

*C*_net_ is a measure of the presence of densely connected clusters within the network. A large *C*_net_ is associated with efficiency of local information transfer in addition to local fault tolerance, broadly meaning that even if vertex *i* fails, its neighbors remain connected (Bullmore and Sporns, 2009).

Often, there are several alternative paths between two vertices *i* and *j*. The path that requires the minimum number of edges to traverse between *i* and *j* is known as the shortest path or geodesic distance, *D*_*ij*_. In a weighted graph, the weights of edges are taken to be inversely proportional to distance in the computation of the shortest path: That is, higher weights correspond to shorter geodesic distances and vice versa. The average minimum path length of a network, *L*_net_, sometimes referred to as the characteristic path length, represents the average of the shortest paths between all pairs of vertices in the network:

(11) $$L_{\text{net}}=\frac{1}{N(N-1)}\sum_{i\;\neq \;j\;\in \;V}D_{ij},\text{ where }D_{ij}\propto \frac{1}{W_{Eij}}$$

*L*_net_ is a global property that measures the overall navigability of a network: A small *L*_net_ is consistent with a well-integrated network capable of efficient parallel information transfer (Barabasi and Oltvai, 2004; Bullmore and Sporns, 2009).

### Calculating sigma

Network organization exists on a continuum with two extremes, perfectly ordered and perfectly random graphs. Watts and Strogatz showed in their algorithm that small world networks have *L*_net_ comparable to, but *C*_net_ much larger than, those of perfectly random graphs. Hence, the small worldness of a network can be expressed in a convenient single-value parameter, the ratio of its clustering coefficient to its path length with both metrics normalized by their corresponding values in an equivalent random graph. Formally this is written as:

(12) $$\sigma =\frac{\gamma }{\lambda }=\frac{C_{\text{net}}/C_{\text{net}<\text{rand}>}}{L_{\text{net}}/L_{\text{net}<\text{rand}>}}$$

As expected, sigma is larger than one for networks exhibiting small world properties (Humphries et al., 2006).

The node degrees of a theoretical random network follow a Poisson distribution which may differ from the degree distribution of the original graph. For this reason, calculation of sigma should be performed with a surrogate random graph configured with the same *k*_net_ and *P*(*k*) as the graph of interest (Sporns and Zwi, 2004). Thus, for each graph (*G*_*A*_ and *G*_*W*_), we generated surrogate random graphs (*G*_*A*<*rand*>_ and *G*_W<rand>_) by randomly reshuffling the paths of node *i* to another nodal location and repeating this process for each node *i* = 1.2, 3 … *N* until the graph was completely randomized but *k*_net_ and *P*(*k*) were preserved. This randomization process went through 25 iterations to compile each surrogate random graph.

### Selecting thresholds

Applying a single arbitrary threshold τ to the association matrices *M* would confine our analysis to the properties of the resulting set of graphs. We therefore methodically calculated graph theoretical metrics over a range of thresholds. The cells of our association matrices *M* contained coherence values between 0 and 1; hence, when τ = 0, all edges are present in the graph (*E*_max_ = *N*×(*N*−1)/2 = 4, 122, 756 in our graphs), and when τ = 1, no edges exist in the graph (*E*_min_ = 0).

One can apply the same threshold (for example τ = 0.5) to each graph. Although straightforward, this process results in a set of graphs with varying numbers of edges. In order to genuinely compare topological and functional characteristics between graphs, it is important to ensure that all graphs contain the same number of edges at a given threshold. This can be achieved using the threshold-dependent cost factor *K*(τ) of a graph, which is defined as the number of existing edges divided by the maximum potential number of edges, 0 ≤ *K*(τ) = *E*(τ)/*E*_max_ ≤ 1 (Latora and Marchiori, 2001; Achard and Bullmore, 2007).

Low values of *K*(τ) result in sparse graphs in which the number of false-positive connections is minimized (Breakspear and Terry, 2002). On the other hand, very low values of *K*(τ) lead to either fragmentation of graphs into sub-graphs when nodes become disconnected or conditions in which small world properties are no longer estimable because the average degree becomes less than the log of the number of nodes (*k*_net_ < ln(*N*) = 7.96 in our graphs).

**Figure 1:**
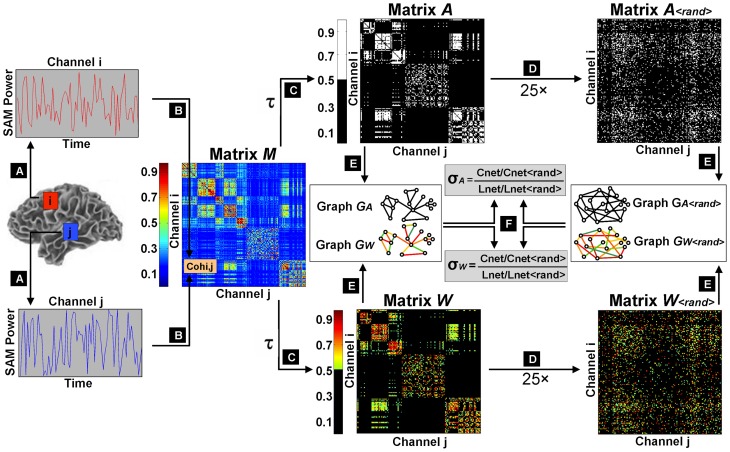
**Schematic flowchart of small world index calculation**. The first step was the extraction of filtered, source-localized magnetoencephalographic time series from 2872 voxels across the brain **(A)**. Magnitude squared coherence was then computed as an estimation of functional connectivity between all pairwise combinations of virtual channels to construct an association matrix M **(B)**. Sliding thresholds τ were used to derive adjacency matrices A and weighted matrices W, with cost factors K(τ) ranging from 0.04 (sparsest matrix: 4% edges retained) to 0.7 (densest matrix: 70% edges retained) **(C)**. A randomization procedure was iterated 25 times on the thresholded weighted and unweighted matrices to obtain surrogate random networks with random connectivity organizations but preserved knet and P(k) **(D)**. Original matrices and their randomized counterparts were then converted into undirected graphs **(E)**, and small world parameters (Cnet and Lnet) were computed for the graphical models of functional brain networks GA and GW, and normalized by their equivalent values (Cnet<rand> and Lnet<rand>) in randomized graphs GA<rand> and GW<rand>, to determine small world indices σA and σW **(F)**.

We therefore selected our lowest value of *K*(τ) to fulfill two criteria: The largest connected component had to contain at least 99% of nodes and *k*_net_ had to be larger than 7.96. We found that the limiting constraint occurred just below *K*(τ) = 0.04 when graphs from several subjects showed disconnection of >1% nodes. Therefore, we calculated small world metrics over 33 separate thresholds of *K*(τ), ranging from 0.04 to 0.3 (in 0.01 intervals) and 0.4 to 0.9 (in 0.1 intervals). For a schematic representation of small world calculation and threshold selection, refer to Figure 1.

### Calculating physical connection distance

The topological distance between brain areas, emphasized in graph theory, is often related to the topographical (physical) distance between brain regions, and minimization of cortical wiring is typically observed in small world brain networks (Bullmore and Sporns, 2009).

We estimated the connection distance of an edge, *d*_*ij*_, as the Euclidean distance between the three-dimensional centroids of voxels *i* and *j* in standard stereotactic space: *d*_*ij*_ ~ (*x*_*i*_ − *x*_*j*_)^2^ + (*y*_*i*_ − *y*_*j*_)^2^ + (*z*_*i*_ − *z*_*j*_)^2^. The mean connection distance (*d*,mm) of each brain network was defined as the average of the connection distances over its edges. Therefore, unlike the topological and dimensionless metrics we used in graph theoretical measurements, the connection distance describes a spatial property of the network (axonal length) and has units of millimeters.

### Producing degree distribution plots

Degree distribution analysis was performed on the sparsest weighted network (*K* = 0.04), as this strict threshold eliminates weaker noisy connections (Achard et al., 2006) while remaining valid for small world network analysis. Previous studies suggest that small world brain networks may be fitted for three candidate models based on the frequency distribution of their node degrees: exponential, power-law, and exponentially truncated power law (Amaral et al., 2000), which we briefly describe.

Random graphs follow an exponential degree distribution: *P*(*k*) ~ *e*^−α*k*^ (Bassett and Bullmore, 2006), indicating an absence of hubs since the majority of nodes have degrees similar to k_net_ (Barabasi and Oltvai, 2004). Most networks in the real world, however, have degree distributions that strongly deviate from those of random models.

Complex systems, such as the World Wide Web, approximate a power-law degree distribution: *P*(*k*) ~ *k*^−α^ (Barabasi and Albert, 1999; Barabasi and Oltvai, 2004). These networks are called scale free because they demonstrate the coexistence of nodes with largely different degrees (scales).

Physically embedded networks, such as transportation systems, have nodes with finite capacities that reach their maximum degree when they can no longer physically accommodate more connections. These networks follow an exponentially truncated power-law distribution: *P*(*k*) ~ *k*^α−1^e^k/kc^ (Guimera et al., 2005; Bullmore and Sporns, 2009). The form of their degree distribution suggests that they are likely to have a stronger hub presence than in comparable random configurations but a lesser hub presence than in comparable scale free networks (Bassett and Bullmore, 2006).

We evaluated goodness of fit for the three statistical models described above using Akaike information criterion, a method that accounts for the differences in degrees of freedoms (Achard et al., 2006). In this process, we used a cumulative distribution to reduce the effects of noise (Strogatz, 2001; Gong et al., 2009).

### Statistics

Statistical comparison of brain metrics between the two groups was performed using a two-sample *t*-test at each *K*(τ) using the voxel-wise 3dttest command from AFNI (Cox, 1996; He et al., 2006; Micheloyannis et al., 2006; Liu et al., 2008).

## Results

### Clustering coefficient and characteristic path length

**Figure 2:**
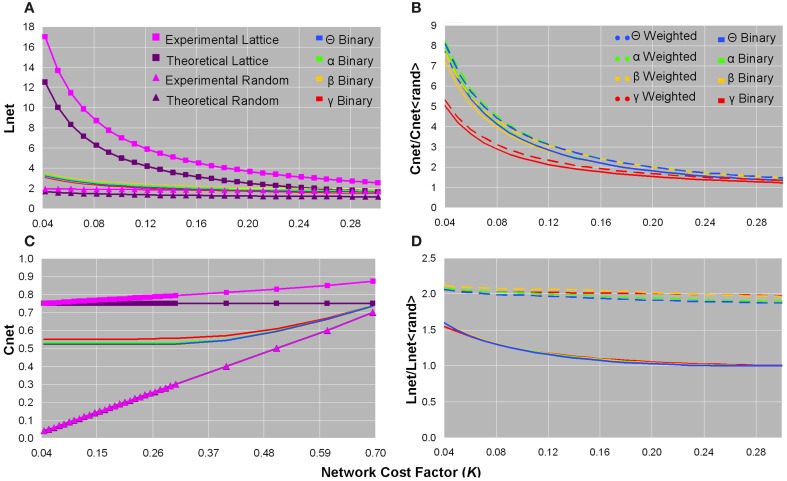
**Plots of the characteristic path length (A), normalized clustering coefficient (B), clustering coefficient (C), and normalized characteristic path length (D) as a function of network cost factor for averaged healthy control datasets**. In each bandwidth, as the network cost increased, the binary Lnet decreased **(A)** and the binary Cnet increased **(C)**. Binary Lnet and Cnet were both intermediate between their corresponding values in lattices and random networks configured with the same number of edge connections and calculated both theoretically and experimentally **(A,C)**. Note that **(C)** is presented until a larger cost factor as a means to demonstrate that the binary networks remained intermediate between ordered and random configurations until convergence. Normalized binary and weighted clustering coefficient values decreased as more edges were added and were smaller in the γ bandwidth than in the other frequency bands **(B)**. Unweighted and weighted normalized path length values decreased in denser networks, and binary graphs showed smaller values than those of weighted graphs **(D)**. For visualization purposes, only data from healthy controls are plotted since these values did not significantly differ in patients.

We concentrated on binary, rather than weighted, *C*_net_ and *L*_net_ brain network values to compare them to their corresponding values in ordered and random networks (Watts and Strogatz, 1998; Stam et al., 2009). The binary *C*_net_ increased as the network became denser, and was less than experimental and theoretical (*C*_net_ = 0.75) values of same-cost lattices, but greater than experimental and theoretical (*C*_net_ = *k*_net_/*N*) values of same-cost random networks (Figure 2C). The binary *L*_net_ decreased as the network contained more edges, and was also less than experimental and theoretical (*L*_net_ = *N*/2*k*_net_) values of regular networks, but greater than experimental and theoretical [*L*_net_ = ln(*N*)/ln(*k*_net_)] values of random networks configured with the same edge connection densities (Figure 2A). Although binary functional brain networks were topologically intermediate between ordered and random structures, in the sparsest networks, binary *C*_net_ was closer to that of a regular network and binary *L*_net_ was closer to that of a random network (Figures 2A,C). All of these trends for binary *C*_net_ and *L*_net_ values were observed in each frequency bandwidth (Figures 2A,C), and healthy controls and patients did not show significant differences in any binary *C*_net_ and *L*_net_ values.

### Normalized clustering coefficient and normalized characteristic path length

For all bandwidths, binary and weighted normalized *C*_net_ and *L*_net_ increased as the number of network edges decreased; however, normalized *C*_net_ values increased considerably more than normalized *L*_net_ values (Figures 2B,D). In sparse networks, the γ frequency band showed smaller normalized binary and weighted *C*_net_ values than the other bandwidths (Figure 2B). Compared to weighted networks, binary networks showed smaller normalized *L*_net_ values as a function of network cost (Figure 2D). No significant differences were found in normalized *C*_net_ and *L*_net_ values between healthy controls and patients.

### Small world index

**Figure 3:**
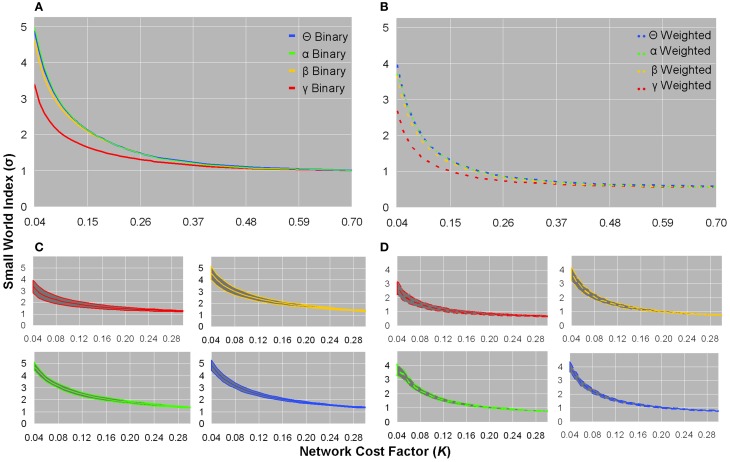
**Small world indices for healthy controls**. Plots of binary graph average small world index **(A)** and standard deviation **(C)**, and weighted graph average small world index **(B)** and standard deviation **(D)**. In binary and weighted sparse graphs, the γ bandwidth showed smaller σ-values than the other bandwidths **(A,B)**. Binary σ-values were larger than weighted σ-values as a function of network cost, and binary σ-values monotonically declined toward an asymptotic value of ~1, whereas weighted σ-values monotonically declined toward an asymptotic value of ~0.56 **(A,B)**. In sparsely connected networks, both binary and weighted graphs showed σ >> 1 **(A,B)**. The standard deviation of σ-values showed little inter-subject variation **(C,D)**. The σ-values from healthy controls were not significantly different in patients.

Small world indices did not significantly distinguish between healthy controls and patients, and all subjects showed σ-values greater than unity in low *K*(τ) networks in each frequency band of binary and weighted networks (Figures 3A,B). Although the standard deviation of σ-values was largest in the sparsest networks, inter-subject variation was minimal (Figures 3C,D). The γ frequency showed smaller σ-values compared to the other frequencies in both weighted and unweighted graphs (Figures 3A,B). For a given bandwidth and cost factor, binary networks showed larger σ-values than weighted networks (Figures 3A,B). As networks became denser, σ-values monotonically decreased toward an asymptotic value of ~1 in binary networks and ~0.56 in weighted networks (Figures 3A,B).

### Physical connection distance

**Figure 4:**
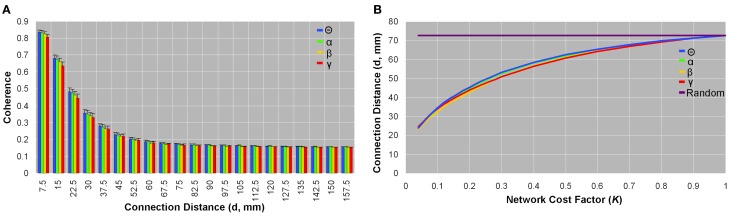
**Organization of physical connection distances between brain voxels**. Coherence as a function of connection distance **(A)**, and connection distance as a function of network cost **(B)** in healthy control data. Coherence between spatially close voxel pairs was on average higher than spatially remote voxel pairs; this pattern was observed in each frequency band **(A)**. Small standard deviation bars indicate that the average coherence between voxel pairs placed in bins as per physical connection distance varied little between subjects **(A)**. Similarly, the average physical connection distance between all voxel pairs was smaller in sparse networks than in dense networks, and this trend was seen across all frequencies **(B)**. In the most sparse brain networks (*K* = 0.04), the mean connection distance was about one-third that of comparable random networks (d ~ 72.8 mm) **(B)**. Patients did not significantly differ from healthy controls in any parameters of physical connection distances.

Networks from all bandwidths showed larger coherence values, on average, in voxel pairs that were physically closer than those that were more physically remote (Figure 4A). There was little variation between subjects in the average coherence between voxel pairs grouped by physical connection distance (<7.5 mm, 7.5–15 mm, 15–22.5 mm, etc.) (Figure 4A). Likewise, as network sparsity increased, the mean connection distance (*d*,mm) decreased: In the sparsest brain network (*K* = 0.04), mean connection distance was three times smaller (*d* ~ 23.7 mm) than it would be in a comparable random network (*d* ~ 72.8 mm) (Figure 4B). Functional brain organization of physical connection distances did not significantly differ between patients and controls.

### Degree distribution

**Figure 5:**
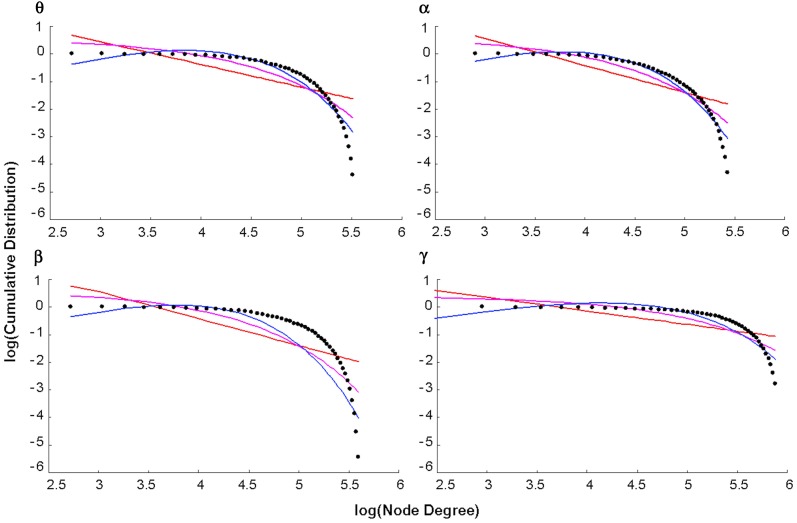
**Degree distributions**. Plots of the log of the cumulative distribution of degree, log(P(k)), vs. the log of degree, log(k), for healthy control group maps in each bandwidth. The black dots represent observed brain data, the pink line is the best-fitting exponential, the red line is the best-fitting power law, and the blue line is the best-fitting exponentially truncated power law.

The log-log plot of the cumulative distribution of functional brain network degrees decayed most as an exponentially truncated power law in all cases except the beta control group (Figure 5). In general, average brain networks for healthy controls and patients across each bandwidth showed the most negative AIC value for the exponentially truncated power law (range: −74.7 to −61.9, excluding the beta control group) and the least negative AIC value for the power law (range: −25.5 to −49.0) (Table 1).

**Table 1:** **Akaike information criterion for goodness of fit of the three statistical models**.

**Network**	**P(k) ~ k^−α^**	**P(k) ~ e^−α^k**	**P(k) ~ k^α−1^ e^k/kc^**
Controls θ	−32.875	−48.421	−61.901
Patients θ	−38.565	−55.481	−73.344
Controls α	−36.427	−54.340	−71.227
Patients α	−34.081	−49.736	−65.695
Controls β	−25.527	−37.094	−36.239
Patients β	−36.645	−51.687	−64.879
Controls γ	−49.038	−65.111	−74.734
Patients γ	−43.588	−57.906	−66.663

### Post-hoc analysis: non-global measurements

Although graph theoretical global parameters (small world metrics, mean connection distance, and degree distribution) were quantifiably similar to those reported in previous healthy volunteer studies, they did not show significant differences between the functional brain networks of healthy controls and patients. We therefore investigated whether functional connectivity at a less global level would significantly differentiate between the subject populations. It would be impractical for us to explore a range of thresholds for this purpose, and so we concentrated on weighted graphs of the strictest threshold (*K* = 0.04), which we had previously used to examine degree distribution.

First, we explored functional connectivity metrics at the scale of individual brain voxels. To do this, we defined the mean functional connectivity of voxel *i*, *F*_*i*_, as the average coherence it shared with all other voxels in the brain:

(13) $$F_{i}=\frac{1}{N-1}\sum_{i\;\neq \;j\;\in \;V}Coh_{ij}$$

For each subject, we generated a vector *F* [1×2872], where elements represented the mean functional connectivity for the 2872 brain voxels. We saved these vectors as ASCII text files, along with an ASCII list of the 2872 brain coordinates, and converted them into 3D-functional datasets using the “3dUndump” command in AFNI (Analysis of Functional Neural Images) Software (Cox, 1996).

Talairach aligned volumes were computed for each dataset, and 3D-mean maps of healthy controls and patients were computed across each bandwidth. From these group maps, we determined the spatial locations of the fifty voxels with the highest mean functional connectivity, as these brain regions might represent hubs that play important roles in network organization (Van den Heuvel et al., 2008). It was important to determine the anatomical location of these functional hubs: Our findings from the main analysis that brain graph nodes followed a heavy-tailed exponentially truncated power law degree distribution had suggested that most brain regions have low linkage and are held together in the system by the few regions that have high linkage (the hubs).

We found that brain regions with the largest mean functional connectivity were relatively consistent across frequency bands, and included the caudate, cerebellar tonsil, cingulate gyrus, culmen, claustrum, hippocampus, parahippocampal gyrus, PCC, and thalamus (Table 2). In general, lower frequency (θ and α) maps showed functional hubs predominantly in the culmen and cerebellar tonsil, whereas higher frequency (β and γ) maps showed functional hubs mainly in the PCC. Across all bandwidths, the thalamus served as a key functional hub (Table 2). All of these potential resting-state hubs widely overlapped between healthy controls and patients (Table 3).

**Table 2:** **Hub locations: The fifty voxels with the largest mean functional connectivity per bandwidth**.

	**Healthy controls**				**Patients**			
	**Atlas region**	**Bilateral**	**Left**	**Right**	**Atlas region**	**Bilateral**	**Left**	**Right**
4–8 (θ)	Culmen	20	9	11	Culmen	14	3	11
	Cerebellar Tonsil	14	6	8	Cingulate Gyrus	11	8	3
	Thalamus	7	5	2	Thalamus	10	5	5
	Cingulate Gyrus	5	4	1	Cerebellar Tonsil	6	3	3
	Parahippocampal Gyrus	2	0	2	Posterior Cingulate	3	3	0
	Posterior Cingulate	1	1	0	Parahippocampal Gyrus	3	0	3
	Hippocampus	1	0	1	Hippocampus	3	0	3
8–14 (α)	Culmen	21	8	13	Culmen	13	6	7
	Cerebellar Tonsil	14	5	9	Cerebellar Tonsil	11	3	8
	Thalamus	6	1	5	Thalamus	9	1	8
	Caudate	4	0	4	Caudate	9	1	8
	Parahippocampal Gyrus	3	0	3	Posterior Cingulate	4	4	0
	Posterior Cingulate	1	1	0	Cingulate Gyrus	3	2	1
	Cingulate Gyrus	1	1	0	Claustrum	1	0	1
14–30 (β)	Posterior Cingulate	18	12	6	Posterior Cingulate	17	11	6
	Cingulate Gyrus	13	7	6	Thalamus	15	10	5
	Thalamus	6	3	3	Cingulate Gyrus	8	2	6
	Cerebellar Tonsil	4	2	2	Caudate	5	0	5
	Culmen	4	4	0	Culmen	3	2	1
	Caudate	3	0	3	Parahippocampal Gyrus	2	1	1
	Parahippocampal Gyrus	2	1	1				
30–80 (γ)	Posterior Cingulate	20	13	7	Thalamus	27	16	11
	Thalamus	17	11	6	Posterior Cingulate	13	8	5
	Cingulate Gyrus	7	4	3	Culmen	4	3	1
	Culmen	2	2	0	Parahippocampal Gyrus	2	1	1
	Caudate	2	0	2	Caudate	2	0	2
	Parahippocampal Gyrus	2	2	0	Cingulate Gyrus	2	1	1

**Table 3:** **Voxel clusters with mean functional connectivity differences between population groups (uncorrected *p* = 0.05)**.

**Freq (Hz)**	**Vol (mL)**	**Mean CD**	**Peak CD**	**Peak Coord**			**Peak Reg**	**CM Coord**			**CM Reg**	**Overlapping Reg**			
4–8 (θ)	**15,609**	**0.49 ± 0.01**	**0.69**	**−15,**	**60,**	**−24**	**R. Cul**	**−15,**	**52,**	**−23**	**R. Cul**	**R. CbT, R. FuG, R. MTG, R. STG**			
	6,328	−0.38 ± 0.01	−0.45	15,	−8,	6	L. Cul	15,	−8,	10	L. Cul	L. FuG			
	**4,641**	**0.33 ± 0.01**	**0.41**	**15,**	**45,**	**44**	**L. Prec**	**19,**	**43,**	**44**	**L. Prec**			
	4,641	−0.32 ± 0.01	−0.38	0,	0,	50	L. MeFG	3,	2,	45	L. MeFG	L. Cing, L. MeFG, L. MiFG, L. SFG			
	2,953	−0.24 ± 0.01	−0.30	53,	60,	6	L. MTG	47,	66,	9	L. MTG			
	**2,531**	**0.49 ± 0.03**	**0.60**	**15,**	**68,**	**−16**	**L. Cul**	**18,**	**72,**	**−13**	**L. LiG**			
8–14 (α)	**30,797**	**0.44 ± 0.01**	**0.77**	**−30,**	**53,**	**−16**	**R. Cul**	**−13,**	**55,**	**−13**	**L. Cul**	**R. CbT, R. MTG, R. STG, FuG**			
	15,609	−0.34 ± 0.01	−0.45	0,	0,	36	L. Cing	17,	−19,	37	L. MiFG	L. MeFG, L. SFG			
	9,703	−0.43 ± 0.01	−0.53	−15,	8,	14	R. Tha	−19,	8,	16	R. Tha			
14–30 (β)	**32,484**	**0.40 ± 0.01**	**0.64**	**−8,**	**53,**	**−31**	**R. CbT**	**−26,**	**50,**	**−12**	**R. Cul**	**R. STG, R. MTG, R. FuG**			
	**5,062**	**0.25 ± 0.02**	**0.35**	**−8,**	**68,**	**29**	**R. Prec**	**1,**	**73,**	**35**	**L. Prec**	**R. Cun**			
	**3,797**	**0.44 ± 0.03**	**0.58**	**15,**	**68,**	**−16**	**L. Cul**	**17,**	**72,**	**−13**	**L. Cul**			
30–80 (γ)	**38,812**	**0.30 ± 0.01**	**0.46**	**0,**	**68,**	**14**	**L. PCC**	**0,**	**60,**	**30**	**R. Prec**	**L. Prec, R. PCC, Cun**			
	14,766	−0.33 ± 0.01	−0.40	45,	−15,	14	L. IFG	47,	−4,	18	L. I FG	L. Ins, L. PrG, L. PoG			
	**4,219**	**0.38 ± 0.01**	**0.44**	**−8,**	**53,**	**−31**	**R. CbT**	**−10,**	**48,**	**−34**	**R. CbT**			
	**4,219**	**0.29 ± 0.02**	**0.39**	**15,**	**75,**	**−1**	**L. LiG**	**15,**	**80,**	**−3**	**L. LiG**			
	3,797	−0.26 ± 0.01	−0.29	−15,	−8,	−24	R. IFG	−19,	−8,	−25	R. STG			
	2,953	−0.27 ± 0.02	−0.33	−45,	−30,	6.2	R. IFG	−41,	−31,	3	R. I FG			

*Bold values indicate positive differences, non-bold values indicate negative differences*.

**Figure 6:**
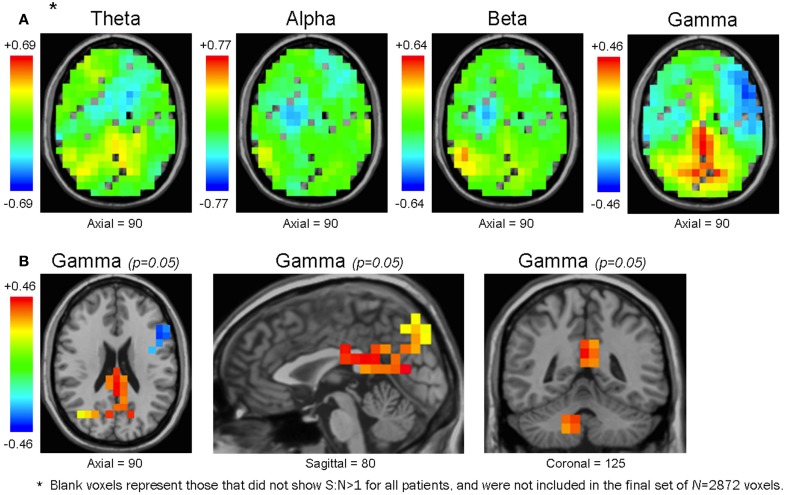
**Maps of the mean functional connectivity difference of healthy controls minus patients**. Unthresholded maps for each frequency bandwidth **(A)**, and maps for the γ band thresholded at an uncorrected *p*-value of 0.05 **(B)**. In the unthresholded maps, some voxels are without values because they did not have S:N>1 for all subjects, and were not included in the original voxel set of *N* = 2872 (see Methodology) **(A)**. In θ, α, and β maps, the voxel cluster with the largest mean functional connectivity difference was positive-valued (i.e., healthy controls had larger mean functional connectivity than patients) and overlapped with the right CbT. In the γ bandwidth, the voxel cluster with the largest difference in mean functional connectivity was also positive-valued but overlapped with the bilateral Prec, Cun, and PCC **(A)**. Unthresholded maps are presented along the same axial plane; therefore, except for the γ bandwidth, they do not show their peak voxels (which would be in the CbT for θ, α, and β maps) **(A)**. Although none of the voxels from any bandwidth survived multiple comparison procedures, at the uncorrected level of *p* = 0.05 the γ map presented trends that accord with our previous resting activation study in which healthy controls showed larger γ SAM power in the bilateral Prec, Cun, and PCC **(B)**.

We also computed 3D-ttest maps between the 3D-functional datasets of healthy controls and patients to determine if any voxels showed significant differences in mean functional connectivity between the populations (uncorrected *p* = 0.05). Across the frequency bands, we found that healthy controls generally showed higher mean functional connectivity in the precuneus, cuneus, PCC, culmen, and cerebellar tonsil, whereas patients typically showed higher mean functional connectivity in the inferior frontal, medial frontal, middle frontal, and superior frontal gyri (Figure 6A, Table 3).

We noted that the largest γ band voxel cluster to survive the uncorrected *p* = 0.05 threshold showed higher mean functional connectivity in healthy controls across the bilateral precuneus, cuneus, and PCC (Figure 6B, Table 3). Interestingly, these brain regions overlapped with the only cluster we identified in a previous resting activation study, in which healthy controls showed higher resting γ SAM power than patients. Consequently, even though none of the voxels from the 3D-ttest maps survived multiple comparison testing, the largest γ cluster at the uncorrected p-level of 0.05 was consistent with our previous resting γ activation findings.

In the last part of the *post-hoc* analysis, we focused on the γ bandwidth to determine whether inter-regional brain pairs showed coherence values significantly different between healthy controls and patients. To accomplish this, we grouped the 2872 brain voxels into 62 brain anatomical seed regions and computed, for each of the 1891 pairwise combinations of brain regions, the average coherence value between all voxels comprising that pair of brain regions. This procedure led to a set of 1891 values for each subject that represented the average γ coherence value between the 1891 brain region pairs.

**Figure 7:**
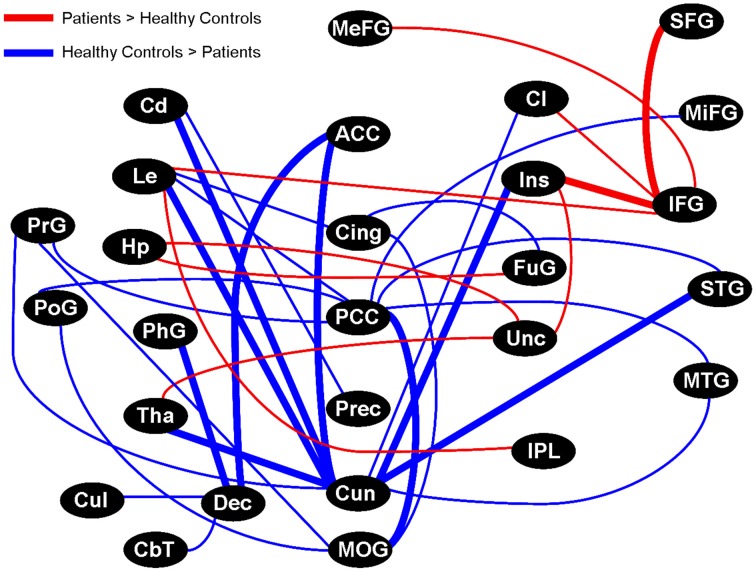
**Graphical visualization of the fifty anatomical pairs with the largest connectivity difference between healthy controls and patients in the γ band**. The pairs represent ~2.5% of the 1891 possible pairs between 62 brainwide anatomical seed regions. The nodes in the above image do not consider brain laterality: However, thick lines are distinguished from thin lines as they represent regions that maintained more than one laterality combination of pairs (for example: R.Cun/R.ACC and L.Cun/R.ACC). Brain regions in which healthy controls displayed higher SAM γ power in our previous study all showed higher inter-regional connectivity in healthy controls, particularly the Cun and PCC, but also the MOG, Cing, and Prec. On the contrary, the IFG and Unc were prominently more connected in patients. Although none of the 1891 pairs passed multiple comparison testing, the top ~2.5% of pairs (uncorrected *p*-value of 0.03) showed trends that are consistent with our previous study.

We arbitrarily focused on the fifty regional pairs (~2.5% of all possible pairs) with the largest γ coherence differences between healthy controls and patients (uncorrected *p* = 0.03). Fifteen of these pairs showed higher γ coherence in patients, and involved several connections to the right inferior frontal gyrus, right uncus, and right insula (Figure 7). In contrast, healthy controls showed higher γ coherence in thirty-five of these pairs, with the most connected brain regions including the bilateral cuneus (18 inter-regional connections) and bilateral PCC (9 inter-regional connections) (Figure 7). Healthy controls also showed higher γ coherence in pairs that included the left middle occipital gyrus, left cingulate gyrus, and left precuneus (Figure 7). All of these brain regions in which healthy controls showed higher inter-regional γ coherence coincided with regions from our previous resting activation study in which healthy controls showed greater resting γ SAM power. Hence, although none of the 1891 inter-regional γ coherence values passed multiple testing corrections, several of the inter-regional pairs in which healthy controls showed higher γ coherence (at the uncorrected p-level of 0.03) were consonant with our previous findings of increased resting γ SAM power in healthy controls across the posterior portion of the medial parietal cortex.

## Discussion

We found that global parameters of functional brain networks were consistent with those of previous reports, demonstrating small world topologies, near-minimum wiring costs, and non-random degree distributions. We also found that all these metrics were preserved in patients with schizophrenia even in the presence of a brain disorder with putative impacts on complex brain functions and treatment with antispychotic drugs, which may also impact on such functions. It is difficult to place this negative finding into the framework of current literature, as reports have varied on how or even whether graph theoretical approaches distinguish between healthy controls and patients (Reijneveld et al., 2007; Stam and Reijneveld, 2007; Bassett et al., 2008; Bullmore and Sporns, 2009; Van den Heuvel and Hulshoff Pol, 2010).

Importantly, however, we found that instead of chance and randomness, there existed a moderate degree of internal order in networks, which might allow for the optimization between segregation and integration of information processing, yielding highly complex brain dynamics. Recent studies have isolated brain regions that show high levels of functional connectivity during resting conditions (Damoiseaux et al., 2006; Buckner et al., 2008), and this phenomenon might be reflected in the high level of clustering we found in networks of this study (Van den Heuvel et al., 2008). The presence of a short characteristic path length in functional networks here might demonstrate that resting brain topology allows for high navigability and streamlined parallel information transfer between resting-state brain regions (Van den Heuvel et al., 2008). Additionally, compared to random networks, brain networks showed reduced wiring costs, which implies that the resting brain conserves energy and material needed for longer axonal projections, while delivering economical small world properties.

Our data add to the growing evidence that small world properties in brain networks may be detectable fairly independent of the methodological procedures used for network construction. Within our study alone, we found that small world architecture was resilient enough to be discerned across eight networks of distinct preprocessing pipelines (four frequency ranges of binary and weighted arrangements). Moreover, our study represents one of the few efforts to apply graph theoretical mathematics at the source-level, and in so doing, we have added to recent endeavors striving to confirm neural correlates to the resting hemodynamic functional connectivity networks more-often analyzed in fMRI (Brookes et al., 2011a), thereby permitting useful cross-modality comparisons that would be impeded if our study were to be performed at the sensor-level (Hillebrand et al., 2011).

### Source versus sensor space

We acknowledge that analyzing functional connectivity in source-space, as opposed to the more prevalent sensor-space, may affect the resulting brain connectivity patterns. However, we decided to analyze the resting networks using SAM beamforming in order to provide the most simple and consistent design needed to continue our line of thought from our original paper, in which we found localized reduction of SAM power in the “resting condition” in our patients (Rutter et al., 2009). In other words, we did not wish to introduce new methodological variability in our subsequent study, other than that we are now determining whether the original baseline differences related to any network abnormalities.

It is also worth noting that there are several drawbacks to using sensor recordings for network construction: For instance, multiple recording sites often detect signals from common sources due to the configuration of the induced magnetic flux, and as such, several groups have advocated for the use of source reconstruction in connectivity analysis to reduce the resulting confounding effects of field spread and volume conduction (Schoffelen and Gross, 2009; Bialonski et al., 2010), as they may lead to erroneously high estimates of functional connectivity (Hillebrand et al., 2011; Jin et al., 2011). Furthermore, multiple signals from spatially separated brain areas merge at the sensor level, which can culminate in over- and under-estimation of synchronization measurements (de Hann et al., 2011; Hillebrand et al., 2011).

These restrictions on sensor-level approaches have prompted exploration into various directions, one being investigation into the use of functional connectivity research at the source level (Hillebrand et al., 2011), an alternative that simultaneously increases the precision of the anatomical regions being studied (Stam et al., 2007b), which may prove particularly imperative in schizophrenia research as a means to thoroughly elucidate whether and how functional brain interactions are impaired (Hinkley et al., 2010). In light of this, several groups that studied resting functional connectivity networks at the sensor level have recommended that future studies address whether similar findings may be detected at the source level (Stam, 2004; Jin et al., 2011; Becker et al., 2012). Also in relation to our study, the specific use of graph theoretical tools to characterize functional networks may be valid at the source level (Palva et al., 2010; Banerjee et al., 2012), and some authors deem that signal space readings may not qualify as genuine vertices seeing they have no clear relation to underlying sources (Antiqueira et al., 2010).

One advantage of the beamformer method, which we employed in our current study, over other inverse procedures is that the number of sources to be estimated does not need to be defined a priori (Aine et al., 2011). Additionally, studies have positively assessed methods of estimating linear and non-linear interaction of neuronal sources using beamformers (Moratti et al., 2011; Vakorin et al., 2011; Wibral et al., 2011), including SAM (Hadjipapas et al., 2005; Vakorin et al., 2010). One recent group, after confirming spatial agreement between resting functional connectivity estimates derived from MEG and fcMRI, concluded that SAM may represent an effective beamformer to model functional connectivity in source space (Brookes et al., 2011a). And several more groups have used similar methods to those of our current study, by mapping MEG functional connectivity across the resting brain using beamforming (Guggisberg et al., 2008; Hinkley et al., 2010), including SAM (Brookes et al., 2011b; Hillebrand et al., 2011). With that said, however, each source reconstruction algorithm employs its own assumptions that may influence correlation estimates between source-based recordings, and beamforming naturally presents its own disadvantages, including partial cancellation of coherent signals and robust cross-talk problems (Hui et al., 2010), knowing that volume conduction and field spread are not entirely eradicated after moving to source-space (Hillebrand et al., 2011).

We now briefly discuss how we interpret our current findings and how they relate to previous reports.

### Small world metrics

Watts and Strogatz established that small worldness exists in sparse networks that require *N* >> *k*_net_ >> ln(*N*) >> 1. In our analysis, the sparsest binary brain networks (*K* = 0.04) showed *L*_net_ closer to that of random graphs and *C*_net_ closer to that of lattices (Figures 2A,C). The strong clustering and short path length in our analysis might support the integration believed to be necessary for efficient information processing in networks (Stam, 2010; Sporns, 2011). Moreover, when normalized by random networks matched for *k*_net_ and *P*(*k*), the sparsest brain networks showed much higher *C*_net_ than *L*_net_ (Figures 2B,D). However, the calculated clustering coefficient, and hence the small world metric, may both be inflated as a result of the problem of artifactual connectivity decreasing with distance in source-level MEG (Schoffelen and Gross, 2009).

These preliminary results may still suggest that sparse brain networks exhibit small worldness, and were in line with our direct computation that σ >>1 in the sparsest networks of healthy controls for both binary (σ : θ = 4.87 ± 0.42, α = 4.99 ± 0.55, β = 4.61 ± 0.58, γ = 3.38 ± 0.55) and weighted (σ : θ = 3.96 ± 0.34, α = 3.95 ± 0.39, β = 3.66 ± 0.46, γ = 2.67 ± 0.45) computations (Figure 3), of which patients showed similar σ-values. Each individual dataset showed small world properties most saliently in the sparsest networks; however, as expected, when networks became denser, the values of σ monotonically declined (Figure 3).

We originally constructed weighted graphs to determine whether the distribution of weights would affect the small world indices computed in the more-often analyzed unweighted graphs, a procedure suggested by recent studies (Jin et al., 2011). In general, we found that weighted graph metrics were qualitatively similar to binary graph metrics, and still did not distinguish between subject populations. However, a notable difference was an overall increase in normalized *L*_net_ in weighted networks compared to binary networks (Figure 2D), a finding that was also observed in a previous study that compared binary and weighted graphs (Rubinov et al., 2009).

We also initially constructed graphs over a range of frequencies to determine whether small world indices would vary across bandwidths. Although the γ band showed decreased normalized *C*_net_ and σ compared to other bands (Figures 2B, 3A,B), we generally found that small world metrics were preserved across frequency bands and still did not distinguish between healthy controls and patients. This finding is broadly consistent with a previous demonstration of frequency scale invariance in resting healthy brain functional networks derived from wavelet decomposition of MEG time series (Bassett et al., 2006).

Additionally, our observation that the small world index did not distinguish between subject populations is somewhat in accordance with a recent study that derived anatomical networks from MRI cortical thickness measurements, and found no differences in small world values between healthy controls and patients (Bassett et al., 2008). And, our findings that no small world values differentiated the subject populations is in line with another study that also found no differences in overall small world value, clustering coefficient, or path length between subject groups using diffusion tensor imaging (Van den Heuvel et al., 2010). However, we should comment that these projects analyzed anatomical, as opposed to functional, brain networks.

In contrast to our experiment, most studies have reported disrupted small world metrics in resting functional brain networks of patients, although these reports have not been consistent (Reijneveld et al., 2007; Stam and Reijneveld, 2007). Nevertheless, the most replicated observation is that of decreased clustering coefficients in schizophrenia from studies using EEG (Micheloyannis et al., 2006; Sakkalis et al., 2006; Rubinov et al., 2009) and fMRI (Liu et al., 2008; Lynall et al., 2010; Alexander-Bloch et al., 2012; Yu et al., 2012). We suspect that differences in these studies, to the extent that they are not methodological, involve differences in clinical samples, in the MEG physical environment, and in other characteristics that might impact on the mental state of patients during the acquisition of the MEG data.

The first of these EEG studies to explore small world metrics used continuous wavelet transform to characterize γ oscillations from healthy controls and patients performing a working memory paradigm (Sakkalis et al., 2006). The authors reported overall reductions in *C*_net_, and *L*_net_ in the functional brain networks of patients. The next EEG study explored functional brain networks derived from synchronization likelihood estimations of signals collected at rest and during a working memory condition (Micheloyannis et al., 2006). Patients showed reduced σ and *C*_net_ in α, β, and γ bandwidths during both resting and working memory conditions. In the most recent of the EEG studies, patients showed lower *C*_net_ and shorter *L*_net_ in comparison to healthy functional brain networks estimated from non-linear interactions of resting-state scalp EEG data (Rubinov et al., 2009).

Several groups have investigated small world networks in resting schizophrenia using fMRI: In one study, partial correlation analysis was used to estimate functional connectivity, and patient networks showed smaller *C*_net_ and longer *L*_net_ (Liu et al., 2008). A more recent project investigated resting functional connectivity in the 0.06–0.125 Hz range, and found that patients showed decreased σ and *C*_net_ values (Lynall et al., 2010). Another group explored modular organization of brain networks by decomposing fMRI data into independent components, and reported decreased *C*_net_ values in patients (Yu et al., 2012). The most recent fMRI study reported reduced *C*_net_ values in childhood-onset schizophrenia (Alexander-Bloch et al., 2012).

That the brain network organization of schizophrenia has been mostly reported as being less clustered, and sometimes less small-world, has led to the hypothesis there may be “subtle randomization” of brain network topology in schizophrenia (Rubinov et al., 2009). However, several studies have showed that the small world index does not distinguish schizophrenia, and other studies have reported that significant population differences in small world parameters may depend upon study methodological parameters: For instance, a recent EEG study by Jalili and Knyazeva found that patients showed increased σ in α bands but decreased σ in β bands, suggesting that the discriminating capacity of graph theoretical parameters in schizophrenia may be fairly dependent on methodologies and modalities (Jalili and Knyazeva, 2011).

Indeed, a recent study found that patients unexpectedly showed increased *C*_net_ values, as well as increased *L*_net_ values, and the authors noted their findings were not in accord with the often-reported decreased clustering in schizophrenia (Yu et al., 2011). Our current study also did not find significant decreased clustering in schizophrenia, and this relatively unexpected finding may be partially due to the fact that we analyzed the brain networks using MEG, instead of EEG and fMRI used in previous studies. As noted above, it may also reflect differences in the mental state of our patients during the MEG procedure in comparison to that of other studies, which could be determined by various uncontrolled and uninterpretable factors (Morcom and Fletcher, 2007).

### Physical connection distance

In light of the fact that longer wiring expends more physical energy (Cherniak, 1994), it has been suggested that the conservation of wiring cost might reflect selection pressures on the evolution of brain networks (Bullmore and Sporns, 2009), and that small world topology might represent an economical layout to minimize axonal volume while maximizing brain complexity (Bassett et al., 2006). As such, spatially close brain regions have a higher probability of being connected than spatially remote regions (Hellwig, 2000; Averbeck and Seo, 2008).

In our analysis, coherence value was negatively correlated with Euclidean distance in functional brain networks (Figure 4A), and the sparsest networks showed mean connection distances one-third that of comparable random networks (Figure 4B). These measurements did not differ between healthy controls and patients, which suggests that all brain networks share the organizational principle of reducing wiring costs, compared to random graphs, especially in the sparsest brain networks that demonstrate the largest σ-values. However, we note that it is difficult to determine whether or how much of the observed reductions in wiring cost can be attributed to the fact that source reconstruction may underestimate true long-distance interactions (Stam and van Straaten, 2012).

One previous study found that both healthy and schizophrenic cortical networks showed less wiring cost than expected in random topologies, and this trend was observed in each classical division of cortex (multimodal, unimodal, and transmodal) (Bassett et al., 2008). Compared to healthy controls, however, people with schizophrenia showed significantly increased mean connection distance in the multimodal networks, an observation that the authors interpreted to mean less wiring efficiency. The same group recently found that task performance in healthy controls and patients during a working memory task was proportional to global cost efficiency in β band MEG networks (Bassett et al., 2009). These findings, combined with our current findings, are in line with the hypothesis that low connection cost might also mean higher efficiency of information transfer in complex brain networks.

With that said, however, the fact that mean connection distance did not distinguish subject populations in our study, and particularly in the orientation that patients would show increased mean wiring cost, a trend that was recently reported in resting fMRI studies of childhood schizophrenia (Alexander-Bloch et al., 2012), may be somewhat inconsistent with previous reports; however, the diverse methodologies employed between the studies and the variable environmental and clinical characteristics may again be partially responsible for these discrepancies.

### Degree distribution

Previous studies of healthy functional brain networks at rest have reported degree distributions that either conform to power-laws or exponentially truncated power-laws. An explanation for divergent findings of scale-free properties is currently unavailable (Bullmore and Sporns, 2009). However, it is notable that truncated power law degree distributions have been more often reported in regional-resolution studies (Achard et al., 2006; Bassett et al., 2006; Lynall et al., 2010), whereas pure power-law scaling have mostly been more often reported in voxel-resolution studies (Eguiluz et al., 2005; Van den Heuvel et al., 2008; Tomasi and Volkow, 2011a). Moreover, resting-state studies that constructed degree distributions at both voxel and regional based resolutions have observed that the higher the resolution, the closer the fit changes from a truncated to a full power law (Hayasaka and Laurienti, 2010).

We found evidence for truncated power-law degree distributions in functional brain networks across all MEG frequency bands in both health and schizophrenia, with the exception of healthy beta band (Figure 5, Table 1). This finding is broadly consistent with previous reports on the topic: One study reported that truncated power-law degree distributions in healthy functional networks were preserved across MEG frequency scales (Bassett et al., 2006). Other studies have confirmed the existence of truncated power-law scaling for both healthy controls and patients in both structural (Bassett et al., 2008) and functional networks (Lynall et al., 2010). However, our study provides unique evidence, although not the first that node degrees of functional brain networks can be best-fitted to truncated power-laws at the voxel-based level. One potential explanation for this discrepancy is that we used SAM prior to the construction of our functional networks, and this preprocessing procedure might have affected the form of the degree distribution differently than studies that did not use source localization techniques (Bullmore and Sporns, 2009).

The current study, along with previous studies, suggests that functional brain networks allow for the emergence of hubs. The presence of highly connected hubs in the default network may be of central importance in intelligence and consciousness (Van den Heuvel et al., 2009; Douw et al., 2011; Soddu et al., 2011). With that said, however, truncated power-law distributions often represent networks that are physically restrained so that the development of very highly connected hubs is less probable than a power-law would anticipate (Amaral et al., 2000; Strogatz, 2001). Therefore, our observation of truncated power-law scaling in networks could likewise reflect the metabolic constraints of maintaining long-range brain connections (Xulvi-Brunet and Sokolov, 2002), or an upper-limit on the number of connections that brain regions can accommodate (Albert and Barabasi, 2000).

### Post-hoc analysis: 3D-mean functional connectivity maps

We determined the spatial location of potential brain hubs, defined as voxels holding the largest mean functional connectivity, implied to be present in the networks since their degrees decayed as exponentially truncated power laws. Healthy controls and patients showed similar functional hub regions: The most notable regions included the bilateral culmen and cerebellar tonsil in the θ/α bands, the bilateral PCC in the β/γ bands, and the bilateral thalamus across all bands, and to a lesser extent, bilateral cingulate gyrus, caudate, and parahippocampal gyrus (Table 2).

Surprisingly, we did not find the precuneus to serve as a hub, even though it has been regularly reported as a functional and structural resting-state brain hub (Achard et al., 2006; Hagmann et al., 2008; Iturria-Medina et al., 2008; Van den Heuvel et al., 2008; Bullmore and Sporns, 2009; Gong et al., 2009), and accounted for the most resting γ SAM power in our previous resting activation study for all subjects (Rutter et al., 2009).

Despite that, many regions overlap with hubs from previous reports: One group first analyzed healthy brain networks using low frequency oscillations of BOLD fMRI time series, and pinpointed the bilateral posterior cingulate and thalamus to be major functional resting hubs (Van den Heuvel et al., 2008), then analyzed healthy brain networks using DTI, and found bilateral caudate, cingulate, precuneus, and thalamus to be major structural hubs (Van den Heuvel et al., 2010).

More recently, another group thoroughly investigated the variability of resting MRI functional connectivity hubs using an impressive sample size of about 1000 subjects across various institutions. They employed a density mapping technique that does not require a priori hypotheses of preselected seed regions, and found that, for both long- and short-range connectivity assessments, the strongest hub consisted of the ventral precuneus and posterior cingulate, and was functionally connected to the cerebellum and thalamus, two major subcortical hubs also found in the current study. Additionally, the authors noted that the most connected hub was functionally linked to the default mode network, and proposed that their findings may be consistent with studies reporting aberrant resting functional connectivity in schizophrenia considering that the related hub regions are known to heavily impact normal and altered states of consciousness (Tomasi and Volkow, 2011a,b).

### *Post-hoc* analysis: 3D-Ttest functional connectivity maps

We produced 3D-Ttest maps of mean functional connectivity to determine whether this metric, in contrast to graph theoretical parameters, would distinguish healthy controls and patients at rest. The resulting ROIs from the 3D-Ttests (Table 3) were broadly similar to the three γ ROIs we isolated in our SAM power 3D-Ttests from our first study, in which patients and unaffected siblings showed reduced SAM power in the posterior medial parietal cortex, and unaffected siblings showed increased SAM power in the superior, medial, and middle frontal gyri (Rutter et al., 2009).

We found that mean functional connectivity was reduced in patients across the posterior medial parietal cortex (θ, β, and γ), and increased in patients across superior, medial, and middle frontal gyri (θ and α) (Figure 6, Table 3). Furthermore, our γ cross-regional analysis complemented these results, and patients showed pronounced interregional connectivity decrease in the medial parietal cortex seed regions, as well as interregional connectivity increase in the frontal gyri seed regions, with the exception of the middle frontal gyrus (Figure 7).

Although these results must be viewed with considerable caution, as none survived statistical correction, we suggest that the previous statistically-significant differences in resting γ SAM power in the same subjects may in the future be robust enough to be discerned with improved functional connectivity measurements. It should be noted that our findings should be viewed with caution for the additional reason that we performed many comparisons in this study as a whole, thus increasing the overall potential for multiple comparison errors. Overall then, it may be ideal to view our study as one that did not address absolute significance between patient populations, but one that did provide evidence that magnitude squared coherence may not be quite strong enough to differentiate functional networks.

With that said, in both our previous and current study, the brain regions most dissimilar between subject populations coincided at least to some degree with components of the default mode network, a brain system that was implicated after multiple neuroimaging approaches converged on what has been proposed as anatomical correlates of the resting state (Buckner et al., 2008). In its strictest definition, the default network consists of the medial prefrontal cortex (MPFC) extending to the ventral anterior cingulate, the PCC extending to the precuneus, and the lateral parietal cortex (Buckner et al., 2008). These brain regions, in addition to being consistently coupled in normal subjects from the so-called resting state, have also been implicated in various states of introspective mentation, including autobiographical memory, theory of mind, and moral decision making, as well as various states of spontaneous cognition, including momentary lapses in attention, and several of these processes may be altered in people with schizophrenia (Buckner et al., 2008).

In line with this, abnormal effects on gray and white matter have been extensively reported in schizophrenia, and the strongest effects have been found in frontal and parietal regions that overlap with the default mode network (Van den Heuvel and Hulshoff Pol, 2010). Likewise, studies have consistently observed aberrant resting functional connectivity in schizophrenia between the medial frontal cortex and the precuneus, key regions of the default mode network (Van den Heuvel and Hulshoff Pol, 2010). Unfortunately, most of these findings, including the anatomical findings with MRI, are also associated with antipsychotic drug treatment, making differences observed between patients and controls based on these various neuroimaging measurements very difficult to interpret.

Moreover, while it has been uniformly reported that default network brain regions are aberrantly coupled in patients during so-called rest and task, reports have been less consistent regarding *how* this aberrancy is characterized across these brain regions. Several studies have suggested an overactive connectivity between brain regions comprising the default network in schizophrenia (Zhou et al., 2007), and in some cases this increased connectivity has been correlated to worsened task performance in patients (Harrison et al., 2007), and increased severity of disease symptoms in patients (Garrity et al., 2007) and first-degree relatives (Whitfield-Gabrieli et al., 2009). In contrast, other studies have reported evidence of decreased connectivity between default brain regions in patients (Lui et al., 2010; Rotarska-Jagila et al., 2010), and in some cases this reduced connectivity has been correlated to increased disease symptoms in patients (Bluhm et al., 2007) and people at genetic high risk for schizophrenia (Jang et al., 2011).

Along the same lines, while some resting analyses of schizophrenia have presented evidence of hypoconnectivity in the frontal regions of the default network, other resting studies have reported hyperconnectivity in the same regions, which may be in direct opposition to the hypofrontality hypothesis in schizophrenia.

Authors have proposed that the inconsistency of these findings may be related to neuroimaging modality differences; task design differences, especially regarding pure resting scans vs. resting periods extracted from task performance, as these situations may affect the likelihood that subjects can reach an appropriate level of internal thought; data analysis differences, especially regarding whether or not ROI seeds are preselected; and participant selection differences, especially regarding whether or not various subtypes or stages of schizophrenia may experience the resting condition differently (Camchong et al., 2011; Jang et al., 2011; Repovs et al., 2011).

Other authors have reported data in which patients showed significantly reduced power in default mode low frequency bins, but significantly increased power in default mode high frequency bins, implying that frequency selection may not only affect, but may also reverse, significant default mode differences between health and disease (Garrity et al., 2007). And some researchers reviewing functional connectivity studies of resting schizophrenia have suggested that hyperconnectivity along one pathway may not necessarily be inconsistent with disconnection along a second pathway, which may have been affected by the altered variance detected in the first pathway, and hence seemingly antithetical relationships may be related (Hoffman and Hampson, 2012).

Taken together, these findings may still substantiate dysconnectivity models of schizophrenia, only now implying that connection alterations are manifested by both quantitative and qualitative changes that vary across the default network, as opposed to any strict global increase or decrease of functional connectivity in resting schizophrenia (Woodward et al., 2011). One recent qualitative change has included the expansion of the default mode network to include less-traditional brain regions that also consistently show differences between resting health and schizophrenia, most notably the inferior frontal gyrus and lateral temporal region, both of which showed trends of aberrancy in our current study (Table 3, Figure 7) (Mannell et al., 2010; Salvador et al., 2010; Skudlarski et al., 2010; Woodward et al., 2011).

Moreover, several studies have detected aberrant cerebellar functional connectivity in resting schizophrenia (Honey et al., 2005; Kim et al., 2008; Becerril et al., 2011). Some authors have noted that the majority of these reports indicate reduced connectivity between the cerebellum and other brain areas, especially the inferior frontal gyrus and thalamus (Collin et al., 2011; Petterson-Yeo et al., 2011). Disruption of this cortical-subcortical-cerebellar circuit in resting schizophrenia has been postulated to relate to impairment in cognitive adaption and coordination in the disease (Bluhm et al., 2007; Collin et al., 2011), and has been found to correlate to more severe disorganization symptoms and less efficient cognitive performance among patients (Repovs et al., 2011). Interestingly, we found that functional connectivity of the cerebellum, as well as the culmen, was reduced in the schizophrenia cohort across all frequencies (Table 3).

In a broader context, the inconsistencies in the literature likely reflect a more generic problem in the interpretation of resting studies in patients with complex behavioral disorders. Since *in vivo* physiologic data presumably reflect the mental activity of the subject, which is what makes it of interest, the assumption that patients with schizophrenia will experience the laboratory environment, whether it be fMRI or MEG, with the same equanimity and “restfulness” as paid, usually experienced volunteers is highly suspect. Thus, interpreting differences in correlated activity patterns during “rest” across subject groups as inherently about the illness biology rather than the immediate illness state may not be justified.

### Future avenues

While evidence for functional connectivity stability in resting fMRI of normal volunteers has been accumulating, the stability of functional connectivity networks using resting MEG has not been as well-established (Jin et al., 2011). Recently, authors have argued that the most logical approach may be to search for consistent results across studies regardless of the technique used for network construction and analysis (Stam and van Straaten, 2012). And several newer studies comparing health and disease by using the MEG resting condition have applied multiple approaches to the same data as a means to determine the reproducibility and robustness of the two results: One study used both magnitude squared coherence and mutual information when comparing health and Alzheimer's (Alonso et al., 2010), and another study used both synchronization likelihood and phase coherence when comparing health and multiple sclerosis (Schoonheim et al., 2011). Other groups are also proposing new methods that may be valuable for inferring functional connectivity, and are evaluating their algorithms using both resting state and naturalistic stimulation MEG data from healthy subjects (Ramkumar et al., 2011), and some groups are developing improved solutions of the inverse problems that may ensure higher signal-to-noise ratios of source-localized functional connectivity MEG (Tanaka et al., 2011).

Previous functional connectivity comparisons of resting health and schizophrenia have produced divergent results, and the reasons are likely numerous. Nonetheless, the majority of studies have produced remarkably convergent results in terms of the *brain areas* themselves that may be the most differentiated between health and schizophrenia during rest, including traditional default network regions (such as the MPFC, PCC, and precuneus), as well as the inferior frontal gyrus, temporal regions, and the cerebellum. Our study provides evidence that less-established MEG approaches to functional connectivity may also detect trends that these same brain regions may be the most aberrant between subject populations at rest.

However, the current study did not produce statistically-significant differences between subject populations. This brings into question whether or not the graphs we derived from MEG are meaningful in relation to brain processes, and particularly whether or not they are underpowered due to the choice of coherence as a measure of functional connectivity in addition to the small number of participants. At this time, it remains difficult to answer this question as MEG source-level connectivity measures may introduce powerful enough artifacts to render the graphs irrelevant (Schoffelen and Gross, 2009), or they may be consistent with whole-brain connectivity studies that have demonstrated consistency between fMRI and MEG results (de Pasquale et al., 2010; Brookes et al., 2011a), thereby providing grounds that these graphs may be relevant to brain processes.

If newer approaches introduced improved capacities to differentiate between population groups, there are several future avenues we could pursue in continuation of the current study. One option would be to analyze functional connectivity networks of unaffected siblings as potential intermediate phenotypes, which was an option available to us in our original resting study after we *did* determine significant differences between health and schizophrenia (Rutter et al., 2009).

Additionally, this study used coherence to quantify oscillatory interdependencies between brain areas, which did not allow us direct inference about the directionality of information flow between brain regions. Another option would therefore be to employ asymmetrical measures, such as Granger and/or partial directed coherence, to construct effective connectivity models and determine whether healthy controls and patients showed different causal association resting measurements. Moreover, although our selection of coherence thresholds is consistent with one of the more common procedures in the literature, our thresholds could instead be determined by parameterless approaches derived from adaptive neighborhood algorithms (Moujahid et al., 2012).

There were several other limitations in the current study, and more detail about these shortcomings can be found in our original report as we used the same datasets (Rutter et al., 2009). It could be beneficial in the future to determine whether and how the resting differences we found between health and schizophrenia could be affected if no patients were on medications, if patient populations were separated by clinical subgroups, and if anxiety ratings and other behavior parameters were subjectively rated by participants before and after the scanning session. However, even with these factors controlled, it may still prove difficult to interpret how these resting differences may be separate from immediate mental states of the subjects (Morcom and Fletcher, 2007).

### Conflict of interest statement

The authors declare that the research was conducted in the absence of any commercial or financial relationships that could be construed as a potential conflict of interest.
